# A Review of Incidence and Related Risk Factors in the Development of Hepatocellular Carcinoma

**DOI:** 10.7759/cureus.49429

**Published:** 2023-11-26

**Authors:** Mahitha Lampimukhi, Tabarak Qassim, Rakshaya Venu, Nivedita Pakhala, Suchita Mylavarapu, Tharindu Perera, Beeran S Sathar, Arun Nair

**Affiliations:** 1 College of Medicine, Rangaraya Medical College, Kakinada, IND; 2 School of Medicine, Royal College of Surgeons in Ireland - Medical University of Bahrain, Busaiteen, BHR; 3 College of Medicine, Saveetha Medical College, Chennai, IND; 4 College of Medicine, Sri Padmavathi Medical College for Women, Tirupati, IND; 5 College of Medicine, Malla Reddy Medical College for Women, Hyderabad, IND; 6 General Medicine, Grodno State Medical University, Grodno, BLR; 7 College of Medicine, Jagadguru Jayadeva Murugarajendra Medical College, Davanagere, IND; 8 Pediatrics, Saint Peter’s University Hospital, Somerset, USA

**Keywords:** carcinogen, alcohol use, cancer incidence, risk factors, hepatocellular carcinoma (hcc)

## Abstract

Hepatocellular carcinoma (HCC) is a primary liver malignancy, ranking as the seventh most common cancer globally and the second leading cause of deaths due to cancer. This review examines the incidence of HCC, its associated risk factors, and constantly changing global trends. Incidence has been noted to be varying worldwide, particularly due to environmental and infectious risk factors. Chronic hepatitis B (HBV) and C (HCV) virus infections, alcohol abuse, aflatoxin exposure, diabetes, obesity, and tobacco consumption are some of the leading risk factors noted. Eastern Asia and sub-Saharan Africa were noted to have the highest disease burden for HCC, with China representing a considerably large majority. On the contrary, the United States reports a lower HCC incidence overall due to improved vaccination programs against HBV; however, with a rising incidence of prominent risk factor in non-alcoholic fatty liver disease (NAFLD), the trend may very well change. Gender disparities were noted to be evident with men experiencing higher rates of HCC compared to women, which may be due to various environmental and biological factors, including alcohol intake, smoking, and androgen hormone levels. Currently, efforts to reduce the overall incidence of HCC include universal HBV vaccinations, antiviral therapies, aflatoxin prevention measures, genetic screening for hereditary hemochromatosis, and early ultrasound evaluation in patients with liver cirrhosis. Understanding these evolving trends and risk factors is essential in combating the rising HCC incidence, especially in Western countries, where risk factors, such as obesity, diabetes, and metabolic disorders, are on the rise.

## Introduction and background

Hepatocellular carcinoma (HCC) is the most common primary malignancy of hepatocytes that occurs in the setting of chronic liver disease. It is the seventh most common cancer and second most common cause of cancer mortality worldwide [1]. Histologically, HCC accounts for 75% of all liver cancers. Hepatitis B virus (HBV) and hepatitis C virus (HCV) account for 80% of HCC globally. In Eastern Asian and most African countries, chronic HBV infection is the leading cause of HCC [2]. HCC also commonly occurs in the setting of oxidative stress caused by diabetes, obesity, alcohol, and toxins, such as aflatoxins.

There is a huge variation in the incidence of HCC in different countries and regions due to the timing and level of exposure to environmental and infectious risk factors [3]. Eighty-five percent of patients with HCC come from low- or middle-resource countries, particularly Eastern Asia and sub-Saharan Africa [2]. Asia has the highest incidence rate, with China accounting for 47% of the world's burden. In a global study conducted by the Global Cancer Observatory (GLOBOCAN) in 2018, Mongolia has been found to have the highest estimated incidence rate, with Nepal and Morocco having the lowest incidence rate [3]. However, a recent report revealed that the incidence of liver cancer in high-risk countries, such as Asia and Italy, is showing a decline, while countries like India, America, and European countries are showing a rise. This new analysis of worldwide trends can possibly be due to the decline in HBV prevalence in Asian countries [3].

In China, the declining incidence rate is due to many factors, but the key reason was found to be the aflatoxin abatement program that permits the dietary replacement of maize with rice [4]. Aflatoxin and HBV synergistically increase the risk of liver cancer. Thus, along with aflatoxin abatement, the HBV newborn vaccination program with a coverage rate of >99.5% in 2017 has also accounted for the declining trend. In Japan and Italy, the decline is accounted for by a decline in HCV prevalence. In the USA, the rise in the incidence rate is due to the recent increase in HCV prevalence with the opioid epidemic [4].

From all of this, we can see that the global epidemiology of HCC shows a changing trend, such as that with increasing HBV vaccination, more effective treatment against HCV and decreasing levels of aflatoxin exposure. A major cause of concern in recent times is the increasing rate of obesity, diabetes, and metabolic disorders. Urbanization and westernization have increased the rate of obesity in both developing and developed countries [4]. With the recent rise in obesity and diabetes mellitus, nonalcoholic steatohepatitis (NASH) in the absence of cirrhosis has become the leading cause of HCC worldwide, which shows that diabetes and obesity are independent risk factors for HCC [5]. Understanding the key cornerstone factors, especially HBV and HCV [6,7,8,9,10] will significantly have an impact in reducing the disease burden of HCC. An understanding of those changing trends is vital in combating the rising incidence in Western countries like America and Europe.

## Review

Global incidence of HCC

Mongolia (Eastern Asia) has the highest reported incidence rate (78.1/100,000) [9]. The prevalence of chronic HCV infection in Mongolia is >15%, which is strikingly higher than the global HCV carrier prevalence, estimated to be about 3% (ranges from 0.1 to 10%) [11]. This high prevalence is attributed to improper sterilization and disinfection of medical and dental equipment that might contribute to the spread of hepatitis viruses. Most HCV infections are caused by genotype 1 (98.8%) in patients with chronic hepatitis, whereas genotype 2 infections is very rare (1.2%). In this regard, improvements in blood safety and the development and execution of strict disinfection and sterilization guidelines for health-related procedures, such as phlebotomy, injection, and dental and surgical manipulations, are required to control the spread of hepatitis viruses in Mongolia [11]. Table 1 describes the aforementioned global incidence with associated risk factors. 

**Table 1:** Global incidence of hepatocellular carcinoma with associated risk factors HCC: hepatocellular carcinoma, ASIR: age-standardized incidence rate, ICMR: Indian Council of Medical Research, AAIR: age-adjusted incidence rate, NAFLD: non-alcoholic fatty liver disease

Country	Incidence of HCC	Any ethnic predominance in that country	Additional comments	Associated risk factors for HCC
India	International Agency for Research on Cancer (WHO): men (0.7 to 7.5), women (0.2 to 2.2 per 100,000 population per year). According to a study conducted on verbal autopsy, in 1.1 million homes, the incidence of HCC among men is 6.8/100,000 and that among women is 5.1/100,000. According to another study, the incidence of HCC in cirrhotic patients in India is 1.6% per year [6].		Based on autopsy data on HCC in India, 0.2–1.9% of autopsy cases had HCC with a higher prevalence of HCC in the southeastern states of India. Based on the ICMR Cancer registry, the average age-adjusted incidence rates (AAIR) for HCC among men is 0.9-7.5 and that among women is 0.2-2.2 per 100,000 population.	Liver cirrhosis, HBV, HCV, alcohol consumption, aflatoxin exposure, diabetes mellitus, non-alcoholic fatty liver disease (NAFLD), smoking, and tobacco use [6,7]
USA	In the USA, the overall incidence of HCC is lower than that in other parts of the world. The age-adjusted incidence rate tripled from 1975 to 2005 from 1.6/100,000 to 4.9/100,000.	Between 2006 and 2010, the incidence rate per 100,000 was the highest in Asians/Pacific Islanders (11.7), followed by Hispanic (9.5), Black (7.5), and finally White (4.2). One study found that the age-adjusted incidence of HCC was highest among Asians (8.4 per 100,000), followed by Black (4.2 per 100,000) and White (2.2 per 100,000). Another study reported a greater age-adjusted incidence of HCC among Hispanic men (3.29 per 100,000) and women (1.23 per 100,000) compared with White men (1.82 per 100,000) and women (0.60 per 100,000).	The incidence of HCC in the United States is expected to continue to increase over the next decade because of peak HCV infection rates.	HCV from unscreened blood transfusions and IV drug use is the major risk factor. HBV accounts for only 10% to 15% of HCC cases in the United States because of widespread HBV vaccination programs [7].
China	The crude and age-standardized incidence rates (ASIRs) were 26.67 and 17.81 per 100,000, respectively.		The incidence and mortality rates were higher in rural areas than in urban areas both in males and females. The less-developed western areas of China showed the highest incidence and mortality rates, followed by middle and eastern areas.	Chronic HBV infection is the dominant risk factor of liver cancer. HBV contributed to about 59.3% of liver cancer cases in China, while only 8.7% can be attributed to HCV. Other risk factors are chronic HCV infection, heavy alcohol consumption, cigarette smoking, and obesity [8].
Mongolia	Mongolia has the highest HCC incidence in the world (78.1/100,000, 3.5 times higher than China).		Ninety-two to 95% of HCC patients in Mongolia are related with HBV and HCV infections, occurring in 115 cases per 100,000 people per year. Mongolia, where approximately 3.2 million people live, has around 2,000 to 2,200 new cases of HCC every year.	The most common etiologies for HCC in Mongolia were HCV infection (46%), HBV infection (34%), coinfection of HBV and HCV (14%), and alcohol (5.6%) [9].
Nepal	The crude incidence of HCC in Nepal is 0.9 and 0.8 per 100,000 in men and women, respectively, and ASIRs are not known.		The mean age of patients with liver cancer in Nepal was 40 years, with male to female ratio of 2:1 in an audit from 1980 to 1987. However, recent trends show a mean age of 59 years (63.2 years in male and 45.7 years in female), with male to female ratio of 3:1.	In all the studies, it was found that HBV was more common than HCV infection as the risk factor for HCC [10].

Examination of risk factors associated with the development of HCC

The WHO Global Hepatitis Report 2017 states that the regions with the highest rates of HBV endemicity are the Western Pacific (6.2% of the population inhabiting there) and African regions (6.1%), followed by the East Mediterranean (3.3%), Southeast Asia (2.0%), Europe (1.6%), and the Americas (0.7%). The age-standardized incidence rates (ASIRs) in Southeast Asia is 13.3 and Africa is 8.4, with Egypt (32.2) and Gambia (23.9) having the highest ASIRs in Africa [12,13]. Tumor suppressor genes are downregulated, and oncogenes are activated as a result of the oncogenic HBV integrating its genome into the host genome [14]. Both somatic and HBV gene mutations have the potential to stimulate the malignant transformation of liver cells. Double mutations in the pre-C region's G1896A and the basal core promoter (BCP) region's T1762/A1764 have been identified as risk factors for HCC [15]. In individuals with chronic HBV and HCC, point mutations, deletions, and insertions frequently occur in the pre-S1 and pre-S2 areas. Hepatic fibrosis or cirrhosis leading to HCC may result from repeated compensatory hepatocyte growth due to persistent inflammatory liver injury (necro-inflammation) [16]. HCC occurs at an earlier stage (mean age is 48 years) in HBsAg-positive individuals affected with hepatitis D virus (HDV) superinfection, whereas the mean age for HBsAg carriers without HDV is said to be 62 years [17]. Premature stop codons result from mutations at specific locations in the S region in patients with cirrhosis and HCC. When the shortened S protein is expressed, the endoplasmic reticulum stress pathway is activated, inducing DNA oxidative damage and genomic instability, all of which contribute to the development of HCC [18]. Tumor suppressor genes are downregulated, and oncogenes are activated as a result of the oncogenic virus HBV integrating its genome into the host genome. Signaling pathways responsible for carcinogenesis are the Wnt/β signalling pathway, P13K/AKT signalling pathway, MAPK/ERK signalling pathway, and oxidative stress pathways [19]. In 1990, there were roughly 210,200 deaths associated with HBV-HCC, whereas in 2010, there were 341,400 deaths. In sub-Saharan Africa, death is likely to occur in people of median age (39.9), whereas in the Western Pacific region, individuals with median age (54.5) exhibit a higher mortality rate [20].

According to the WHO Global Hepatitis Report 2017, infection with HCV affects 71 million people worldwide. The eastern Mediterranean region (62.5 per 100,000 people) and the European Region (61.8 per 100,000 people) have the greatest incidence rates [21], and the most common means of transmission are improper medical injections and the use of illicit injectable drugs. Baby boomers, people born between 1945 and 1965, make up the cohort with the greatest prevalence of chronic HCV infection and, consequently, the highest rates of liver cancer-related mortality [22].

About 34% of HCC cases in the USA are caused by chronic HCV infection [20], which is the primary cause of HCC in Western nations. In contrast to most parts of Asia and Africa, where HBV is the main cause of HCC, HCV is the predominant cause of HCC in the USA, Europe, Japan, and South America [23]. It has been estimated that the prevalence of HCV in Japan is about 3%, and about 85% of the patients with HCC are affected by HCV. Contrarily, the USA has a lower prevalence of HCV (1.8% of the population), but 50-60% of patients diagnosed with HCC had an HCV infection. The earlier start of the HCV epidemic in Japan has an impact on the higher proportion of patients with HCC and HCV compared to the USA, suggesting that the incidence of HCC associated with HCV will continue to increase in the USA [24]. The direct action of HCV viral proteins on cell signaling pathways leads to the development of HCC by the inhibition of tumor suppressor genes, such as retinoblastoma protein, P53 tumor suppressor, or by activating signaling mechanisms that initiate growth and division [25]. Telomerase, reverse transcriptase, tumor protein 53, and β-catenin are the genes that are most frequently involved in mutations that lead to oxidative stress in HCC [26]. Molecular pathways responsible for HCC include the dysregulation of many signal transduction pathways, such as p53, Ras, Wnt/β-catenin, MAPK (mitogen-activated protein kinase), an activator of transcription (STAT), Janus kinase (JAK)/signal transducer, phosphatidylinositol 3- kinase (PI3K)/Akt, hedgehog, TGF-beta, and epidermal growth factor [27,28]. The activation of hepatic stellate cells and proliferation precede the development of fibrosis, which is followed by the occurrence of HCC in PDGF-C transgenic mice. This progression is identical to that seen in human HCC [29].

It was observed that the consumption of alcohol in quantities greater than 80 g/day for at least five years resulted in an increased risk of incidence in HCC [30]. Alcohol accounts for 30% of cases worldwide, which is the leading cause of liver disease linked with HCC. A major epidemiological study that was based on the literature review of the Global Burden of Disease (2015) estimated 854,000 new primary liver cancer cases and 810,000 deaths in 2015, out of which, 245,000 (30%) were associated with alcohol-related HCC [31]. Alcohol accounts for 6% of the cases in the Middle East (Iran), 14% in North Africa (Morocco), and 50-60% in Eastern Europe. Hepatic alcohol-mediated carcinogenesis is due to different factors, such as the toxic effects on proteins and DNA due to the production of acetaldehyde. Increased production of iron-induced reactive oxygen species (ROS) and CYP2E1 leads to the disruption in the function of DNA repair mechanisms and chronic inflammation, factors interfering with the transfer of methyl group and alterations in the expression of a gene [32]. Changes in folate metabolism due to the polymorphism exhibited by the methyl tetrahydrofolate reductase (MTHFR) gene have been linked to the development of HCC in patients with alcoholic liver disease [33].

According to a systematic review of 81 epidemiological studies, cigarette smoking is linked to an increased risk of incidence and mortality of HCC [34]. Heavy smoking leads to the hepatic accumulation of excess iron, which is the cause of fibrosis and HCC. A decrease in the P53 gene was also observed [35]. However, there have been conflicting findings with respect to HCC associated with smoking in Egypt. Some studies revealed that there was no predominant increased risk of HCC in heavy smokers when factors, such as age and gender, were adjusted [36,37]. However, studies conducted in the mid Delta Region of Egypt demonstrated that pesticides and cigarette smoking are the dominant non-HBV and non-HCV-related risk factors due to significant exposure to pesticides and smoking [38].

The chemical compounds associated with the development of HCC can be divided into organic and inorganic compounds. The inorganic compounds include arsenic and cadmium [39]. Organic compounds include vinyl chloride monomer, polyvinyl chloride, and organic solvents like perchloroethylene, trichloroethylene, dioxine-like compounds, polychlorinated biphenyls, N-nitrosamines, and polybrominated phenyls. Compounds like chloral, chloral hydrate used in insecticides, and dichlorodiphenyltrichloroethane (DDT) and O-toluidine used in herbicides and pesticides are also considered risk factors [40]. Arsenic has been regarded as a group 1 carcinogen (International Association of Cancer Registries (IACR)). According to several epidemiological studies, chronic exposure to arsenic revealed pre-neoplastic lesions, abnormality in the functioning of liver, hepatomegaly, cirrhosis, and fibrosis of the liver [41]. Carcinogenicity due to arsenic may be due to oxidative damage to the DNA, epigenetic and genetic mechanisms, apoptosis alterations, irregularities in DNA methylation, instability of genome, and abnormal signalling of estrogen [42,43,44,45]. Cadmium carcinogenicity was found to involve the induction of oxidative stress and inhibition of methylation of DNA, due to which there is a dysfunction of E-cadherin and induction of protooncogenes [46]. According to a study related to endocrine-disrupting chemicals in male mice, the CYP3A11 gene involved in the inflammatory response in the liver gets upregulated upon exposure to DDT [47]. HCC incidence has been reported to increase upon oral dose of O-toluidine administration to male and female rats [48].

HCC incidence varies by gender, with men having the fifth highest rate (7.5%) and women having the ninth highest rate (3.4%) [49]. The estrogen hormone is associated with a decreased risk of injury to the liver due to its role in the suppression of IL-6-mediated inflammation [50]. There is an increased signaling of androgen receptors by testosterone, which plays a vital role in promoting the proliferation of liver cells [51]. Environmental variations in the incidence of gender-based HCC are attributed to factors, such as increased rates of exposure of men to alcohol, smoking, chemical compounds, occupational exposure, and hepatitis than women [52].

Globesity, the increase in the incidence of obesity, is parallel to the increase in the incidence of HCC globally. There was a 39% increased risk of HCC per five-unit rise in body mass index (BMI) (kg/m^2^) in a meta-analysis of 21 prospective studies shown in a study in 2012 that included 17,624 instances of primary liver cancer [53]. In a landmark study by Calle et al., the mortality from liver cancer was 1.9 times higher in obese men with a BMI of 30-34.9% compared to individuals with a normal BMI (18.5-24.9 kg/m^2^) [54]. According to a major cohort research done in the UK, those with an obese BMI of 30 kg/m^2^ had a mortality rate from liver cancer that was about four times greater than that of people with a normal BMI of 18.5-24.9 kg/m^2^ [55]. The negative prognostic effect of obesity on HCC is due to factors, such as reduced HCC surveillance due to poor quality of ultrasound. Obese individuals are also more likely to experience post-surgical complications, such as hepatic decompensation, bile leakage, and wound infections, all of which may result in postoperative mortality [56]. Remodeling of adipose tissue, ectopic lipid accumulation, secretion of proinflammatory adipokines, lipotoxicity, higher levels of insulin, and insulin-like growth factors due to the development of insulin resistance are the factors that eventually lead to chronic inflammation and development of HCC [57].

A case-control study conducted in Italy demonstrated an inverse relationship between the risk of HCC and a diet rich in beta-carotene and linolenic acid [58]. A study on atomic bomb survivors in Japan revealed that consuming isoflavone-rich miso soup and tofu reduced HCC risk by 50% [59]. Cell culture systems and animal models have demonstrated that constituents of coffee, such as cafestol, kahweol, and diterpenes, play a role as blocking agents via modulating enzymes that are linked to carcinogen detoxification [60]. Interestingly, hepatic adenomas can undergo malignant transformation after usage of oral contraceptive pills for a mean duration of 11 years [61], which leads to a discussion regarding the hormonal influences on the development of HCC. According to a meta-analysis of 11 studies, the percentage of autoimmune hepatitis (AIH) patients with cirrhosis ranges from 12% to 83%, while 5-6% of AIH patients develop HCC [62]; however, further clinical research is required to formally establish the relationship between AIH and the development of HCC. 

It has been estimated that globally, 285 million individuals in the world suffer from diabetes, which accounts for about 6.4% of the total population [63]. By 2030, the prevalence is expected to rise to 69% in developing nations and 20% in developed nations. Diabetes can be regarded as a component of metabolic syndrome, which may eventually lead to non-alcoholic associated steatohepatitis (NASH) and HCC. Type 2 diabetics exhibit a phenomenon of an increase in levels of IGF-1 (insulin-like growth factor-1) due to insulin resistance, which can result in carcinogenesis and oxidative stress due to persistent hyperglycemia [64]. Diabetes has been observed to have an association with mutations in p53 gene, thus putting diabetics at a higher risk for HCC [65].

The prevalence of non-alcoholic fatty liver disease (NAFLD) accounts for about 24% globally, with South America reporting the highest rate of incidence of about 31%. The lowest rate of incidence is found in Africa (14%). Incidence rates in the Middle East, Asia, and the USA are 32%, 27%, and 24%, respectively [66]. Reports suggest that about 25% of patients affected with NAFLD shall progress to NASH, out of which cirrhosis has been reported in 20% of the individuals [67]. According to preliminary research, excess fatty acid supply and hepatocellular steatosis increase fatty acid oxidation, which are the factors resulting in increased reactive oxidative stress [68]. According to one study, which examined 1,168 patients who had their livers removed because of HCC, six out of eight individuals with NASH-related HCC did not have cirrhosis [69]. NAFLD with NASH is also responsible for the significant increase in the production of proinflammatory cytokines, pro-oncogenic signals, and epigenetic changes. Patatin-like phospholipase domain-containing protein-3 (PNPLA3) exhibits genetic polymorphism, which results in a higher risk of HCC incidence [70]. Downregulation of carnitine palmitoyltransferase II aids hepatocytes in escaping lipotoxicity, but the accumulation of acyl carnitine leads to the occurrence of malignant transformation [71]. Obesity-associated proteins, such as junctional protein associated with coronary artery disease (JCAD), which is essential for tumor growth, are highly demonstrated in NASH-HCC [72]. Considering the world’s rising obesity and diabetes rates, NAFLD-NASH is an emerging risk factor for HCC that has the potential to contribute to and eventually replace HCV as the primary risk factor for HCC [73].

Alpha-1-antitrypsin deficiency (A1ATD) cannot be regarded as a sole determinant of HCC incidence, but it does occur in combination with other risk factors, such as HCV, cirrhosis, and NAFLD (Table 2) [74]. Mechanisms, such as altered cyclin D1 and melanoma cell adhesion molecule regulation (MCAM), delayed degradation of endoplasmic reticulum protein, and mitochondrial dysfunction, are associated with A1ATD-related HCC [75,76,77]. Hemochromatosis results in an iron overload that promotes the growth of tumors by underlying mechanisms, such as an increased proliferation of cells, damage to the DNA and cell membranes via peroxidase, and increasing ROS levels [78]. The impairment and inhibition of lymphocytic proliferation is seen as a result of iron overload [79]. Cirrhosis occurs in 10-25% of HH patients, and the incidence of HCC is about 8-10% in HH patients [80]. In the absence of genetic hemochromatosis, individuals of African descent have demonstrated a higher risk of HCC in relation to the increased iron levels due to beta-thalassemia [81]. Hence, it is important to carry out surveillance for HCC in all cases that exhibit iron overload.

**Table 2:** Categorization of risk factors contributing to the development of hepatocellular carcinoma HBV: hepatitis B virus, HCV: hepatitis C virus, NAFLD: non-alcoholic fatty liver disease

Category		Identified risk factors for HCC
Infectious	HBV, HCV	
Non-infectious	Chemical compounds (modifiable), alcohol abuse (modifiable), tobacco consumption (modifiable)	
Host-related risk factors	Non-modifiable: gender, ethnicity, autoimmune mepatitis. Modifiable: oral contraceptives, obesity, diabetes mellitus, NAFLD	
Monogenic risk factors	Alpha 1 antitrypsin deficiency, hemochromatosis	
Polygenic	Family history, aflatoxins	

According to an American study, individuals with a first-degree family history of liver cancer are up to four times more likely to acquire the disease than the general population, which may be due to certain shared genetic and environmental variables [82]. Records show that 21.4% of HCC patients in Egypt have first- and second-degree relatives associated with HCC [83]. Aflatoxin B1 is considered a group 1 carcinogen by IARC [84]. Regions like sub-Saharan Africa, China, and Southeast Asia exhibit the highest rate of exposure to aflatoxin. Gene chip analysis of a sample of HCC tumor tissue revealed that exposure to aflatoxin results in a mutation to the p53 gene at the third base of codon 249 [85].

Global trends in mortality rates for HCC

Globally, HCC ranks as the third cause of cancer-related fatalities [2]. An analysis of HCC mortality trends from 1990 to 2014 revealed the following [86]: Northern and Central Europe had an increase in HCC mortality, while a decline in the mortality rates was observed in Southern Europe. In the USA, between 2002 and 2012, HCC mortality increased by 35%, which accounts for 3.1 per 100,000 men; males above the age of 70 seemed to have the highest mortality rates, with males between the ages of 55 and 69 experiencing the highest increase in mortality [87]. Females also displayed similar patterns of trends. A decreasing pattern of trends in mortality was observed in East Asia, although it accounts for about 10-24 deaths per 100,000 men. In Japan, by the year 2020, there was a decline in mortality in men due to HCC (5.4 per 100,000). The reduction in HCC-related mortality in East Asia and Southern Europe can be attributed to the control of HBV and HCV infections. Compared to most European and American countries, death rates in the East Asia region were two to five times higher. Unfavorable factors, such as HBV/HCV epidemics, increased consumption of alcohol, obesity, and diabetes, are responsible for the rising trends in other regions. Early detection of HCC, treatment, and effective management of cirrhosis are the measures that exhibit a significant impact on global mortality patterns.

Discussion

This study focuses on the incidence of HCC in different countries by assessing and providing a general overview on the geographical location and associated risk factors. We have also aimed to demonstrate how the incidence of HCC can be reduced by HBV vaccination, surveillance with an ultrasound, monitoring alpha-fetoprotein (AFP) twice in a year, and a detailed study of infectious/non-infectious/monogenic/polygenic risk factors and their preventions. The role of obesity, NAFLD, and type 2 diabetes mellitus (T2DM) in carcinogenesis has also concurrently been studied.

Risk factor variations in different countries have shown to affect the HCC incidence rates. Countries with limited access to medical supplies, mostly Eastern Asia and sub-Saharan Africa, have a high incidence rate of HCC (85%) [2]. China accounts for 47% of HCC cases globally [3], chronic HBV infection was considered as the main risk factor contributing to 59.3% of liver cancer cases in China, while aflatoxins, cigarettes, obesity, and alcohol consumption also seem to have an impact [8]. Mongolia has been found to have the highest estimated incidence rate (78.1/100,000) [3]. HCV (46%), HBV (34%), and co-infections of HBV and HCV (14%) have been the risk factors that resulted in such a high incidence rate [9]. The increasing cases of HCC in India are related to liver cirrhosis, chronic HBV infection, chronic HCV infection, alcohol consumption, aflatoxin exposure, DM, NAFLD, smoking, and tobacco use [7]. In the USA, the incidence rate is lower than other parts of the world (4.9/100,000), owing to the HBV vaccination programs and NAFLD causing most of the HCC cases [7]. In Eastern European countries, such as Belarus (63% cases), the high incidence of HCC was linked to a high alcohol consumption [31]. HCC incidence rates also show variations according to gender, with a higher incidence among men. Male to female ratio of HCC incidence exceeds 2.5, attributable to the environmental risk factors, such as alcohol intake, smoking, and occupational exposures. Biological risk factors, such as high androgen levels, which increase liver proliferation, and low estrogen levels, which are responsible for the suppression of IL-6, also contribute to this ratio. We reviewed that low-resource countries show high prevalence of HBV. HBV infections can be reduced by universal HBV vaccinations and effective antiviral therapy. Other precautions can be taken, for example, using disposable needles and syringes for procedures, sterilization of endoscopic equipment, using sterile gloves before handling blood and blood products (especially among healthcare workers), and testing blood products for HBV and HCV prior to transfusion.

Early detection of hemochromatosis by genetic screening and serum iron stores can reduce the risk of HCC associated with hereditary hemochromatosis. In Asian countries, aflatoxin was found to be a major HCC risk factor, so prevention of fungal contamination of grains and ground crops can decrease aflatoxin affecting HCC incidence [88]. Early detection of HCC by ultrasonography for cirrhosis patients has shown to reduce the incidence of HCC [88]. NAFLD, which is responsible for the majority of cases of HCC in countries like the USA, can be avoided with the consumption of dietary antioxidants (vitamins C and E, selenium, and coenzyme Q10) and phytochemicals present in fruits, vegetables, medicinal plants, and herbs, among others. The Mediterranean diet (e.g., vegetable oils, fruits, legumes, vegetables, cereals, and fish) has been found to have protective effects against HCC [5]. Type 2 diabetes has an increased risk of HCC. The use of metformin is associated with a decreased risk, while the use of insulin or sulfonylureas may increase the HCC risk. [89] The results of our study must be interpreted in the context of several limitations. Even though we were able to interpret the incidence rate of HCC by countries, gender, and ethnicity, among others, linking them to an exact risk factor(s) was challenging. This study did not evaluate on how having a certain risk factor could potentiate the incidence of HCC when combined with other risk factors. Nonetheless, we can hypothesize that the main risk factors remain to be HBV and HCV infections and NAFLD in most regions and the other risk factors had a lower impact on HCC incidence comparatively. These limitations may provide further directions for future research studies on the incidence and risk factors of HCC.

HCC is the fourth most common cause of death. In this study, we have detailed risk factors, such as cirrhosis, HBV, HCV, NAFLD, alcohol abuse (ethanol >60 g/day), smoking, aflatoxin exposure, hemochromatosis, obesity, type 2 diabetes, and dietary antioxidants, and how they progress into HCC. Preventive measures, such as HBV vaccination, sonography, and AFP monitoring, help in the early identification of the disease.

## Conclusions

HCC remains a tough global health challenge with varying incidence based on geographic locations and evolving risk factors. Risk factors, such as chronic hepatitis B, aflatoxin exposure, and limited healthcare resources, are seen to be prevalent in Eastern Asia and sub-Saharan Africa, whereas increased prevalence of NAFLD secondary to obesity and diabetes has been a constant battle in the Western countries. It is understood from our analysis that the efforts to combat HCC must be multidisciplinary and multifaceted with the need for vaccination programs, antiviral therapies, prevention measures against aflatoxins, and early detection being key cornerstones. The dynamic nature of HCC requires ongoing research and global collaboration to reduce this disease burden, and moving forward, a complete understanding of regional and epidemiological patterns would be necessary to continue making adaptable recommendations to all members of society.
